# Quality of life among advanced cancer patients in Vietnam: a multicenter cross-sectional study

**DOI:** 10.1007/s00520-021-06012-3

**Published:** 2021-02-03

**Authors:** Bui Thanh Huyen, Pham Thi Van Anh, Le Dai Duong, Than Ha Ngoc The, Ping Guo, Pham Van Thuc, Luong Ngoc Khue, Eric L. Krakauer, Richard Harding

**Affiliations:** 1 Department of Palliative Care, University of Medicine and Pharmacy, Ho Chi Minh City, Vietnam; 2 City Children’s Hospital, Ho Chi Minh City, Vietnam; 3 Department of Infectious Diseases, Hai Phong University of Medicine and Pharmacy, Hai Phong, Vietnam; 4 Department of Geriatrics and Palliative Care, University Medical Center, Ho Chi Minh City, Vietnam; 5 Florence Nightingale Faculty of Nursing, Midwifery and Palliative Care, Cicely Saunders Institute of Palliative Care, Policy and Rehabilitation, King’s College London, Bessemer Road, London, SE5 9PJ UK; 6 Hai Phong University of Medicine and Pharmacy, Hai Phong, Vietnam; 7 Administration of Medical Services, Ministry of Health, Hanoi, Vietnam; 8 Department of Global Health and Social Medicine, Harvard Medical School, Boston, USA; 9 Division of Palliative Care and Geriatric Medicine, Massachusetts General Hospital, Boston, USA

**Keywords:** Palliative care, Cancer, Vietnam, Quality of life, Global health

## Abstract

**Purpose:**

Cancer is a leading cause of death in Vietnam. To maximize quality of life (QOL) at the end of life, valid and clinically useful instruments are needed to assess palliative care needs and the effectiveness of palliative care interventions.

**Methods:**

We aimed to (i) determine psychometric properties of the Vietnamese version of the WHO abbreviated quality of life scale (WHOQOL-BREF_VN_) among advanced cancer patients, (ii) measure HR-QOL, and (iii) identify predictors of HR-QOL. We collected demographic, clinical, and HR-QOL data from stage III/IV adult cancer patients at two major Vietnamese cancer centers. We determined the internal consistency (Cronbach’s alpha), construct validity (confirmatory factor analysis (CFA)), and discriminant validity (known-groups comparison) of the Vietnamese instrument. HR-QOL was analyzed descriptively. Multinomial logistic regressions identified predictors of HR-QOL.

**Results:**

A total of 825 patients participated. Missing data were completely at random (MCAR) (chi-square = 14.270, df = 14, *p* = 0.430). Cronbach’s alpha for all items was 0.904. CFA loadings of physical, psychological, social relationship, and environment domains onto HR-QOL were 0.81, 0.82, 0.34, and 0.75, respectively. Prediction of scores differed significantly by functional status (Wilks’ lambda = 0.784, chi-square = 197.546, df = 4, *p* < 0.01, correct prediction = 74.6%). HR-QOL was reported as very bad/bad by *n* = 188 patients (22.8%) and general health as very bad/bad by *n* = 430 (52.1%). Multinomial logistic regression (likelihood ratio test: chi-square = 35.494, df = 24, *p* = 0.061, correct prediction = 62.2%) and the Pearson correlations revealed worse HR-QOL was associated with inpatient status, high ECOG score, and having dependent children.

**Conclusion:**

The Vietnamese version of the WHOQOL-BREF has excellent internal consistency reliability and sound construct and discriminant validity in advanced cancer patients. Advanced cancer inpatients, those with dependent children, and those with poorer physical function appear to have the greatest palliative care needs.

## Introduction

Cancer incidence and mortality are rising and as much as 70% of global cancer incidence may be in LMIC by 2030 [1]. By 2060, an estimated 16 million people with cancer will die annually with serious illness–related suffering, a 109% increase from 2016 to 2060, with the fastest rise occurring in low-income countries (400% increase) [2].

A systematic review of symptoms in people with advanced illness revealed that more than half of advanced cancer patients suffer from fatigue, anorexia, pain, worry, and poor self-reported well-being [3]. Health-related quality of life (HR-QOL) offers a clinically useful outcome measure beyond morbidity and mortality [4]. Integrated early palliative care improves patient- and family-reported outcomes including quality of life, while reducing costs [5–8]. In response to the huge disparity between high- and middle/lower-income countries in access to palliative care and pain relief [9], the World Health Organization resolved that provision of palliative care is a responsibility of all healthcare systems and affirmed that universal health coverage cannot be achieved without access to palliative care [10].

In 2016, cancer was the leading cause of death in Vietnam, with estimated 164,600 new cancer cases and 114,000 cancer-related deaths. Cancer is usually diagnosed at a late stage in Vietnam [11] which lacks adequate cancer treatment facilities [12]. No data on HR-QOL of Vietnamese cancer patients currently exist. Therefore, this study aimed to (1) evaluate the psychometric properties of a common measure of HR-QOL as applied to patients with advanced cancer in Vietnam, (2) measure HR-QOL, and (3) identify predictors of HR-QOL.

## Methods

### Settings and sample

This cross-sectional, multicenter self-report study was conducted in two public tertiary referral hospitals in Vietnam: a cancer center in Hanoi (northern region) and a general hospital in Ho Chi Minh City (southern region). The cancer center has 1800 inpatient beds and receives patients from throughout northern Vietnam. The general hospital is in southern Vietnam and has 700 inpatient beds.

We recruited consecutive inpatients and outpatients with stage III or IV cancer according to the 2010 TNM staging criteria, aged at least 18 years old, with the mental capacity to participate in a self-report questionnaire. A minimum sample size of 660 was needed to determine the prevalence of WHOQOL-BREF items with a confidence level of 99%, margin of error 5%, and expected distribution of 50% using the 2016 estimates of cancer mortality above. Recruitment was stratified by the hospital, with the sample per site proportionate to the number of cancer patients under care within the previous year.

#### Clinical and demographic variables

All data were collected by medical and public health students trained in survey interviewing. Demographic information collected included age, gender, ethnicity, region, education, job status, monthly income, household size, marital status, and number of dependent children. Clinical measures included place of care, primary cancer, metastases, stage, year of diagnosis, prior treatment with radiotherapy or chemotherapy, and Eastern Cooperative Oncology Group (ECOG) [13] performance status.

#### HR-QOL measure

Data on HR-QOL was collected using the Vietnamese version of the World Health Organization abbreviated quality of life scale (WHOQOL-BREF_VN_) [14]. The WHOQOL-BREF_VN_ is a validated, multidimensional instrument of 26 items consisting of 4 domains and two global items: HR-QOL and general health. Each global item is rated with a Likert scale from 1 to 5. The HR-QOL score is computed by multiplying the reported HR-QOL by 4. Thus, each participant has a HR-QOL score of 4, 8, 12, 16, or 20 (interpreted as “very bad,” “bad,” “neither bad nor good,” “good,” and “very good” HR-QOL, respectively). General health is measured in the same way. The remaining 24 items are grouped into four domains: physical (7 items), psychological (6 items), social relationships (3 items), and environment (8 items). The value of each domain is calculated by multiplying the average of all items in that domain by 4, giving a continuous numerical variable ranging from 4 to 20. A higher score represents better patient status.

The original English language WHOQOL-BREF demonstrated good internal consistency reliability (Cronbach’s alpha of domains 0.66–0.84). Confirmatory factor analysis (CFA) revealed a stable factor structure, and it has excellent discriminant validity between ill and well participants. The WHOQOL-BREF_VN_ was found to have excellent internal consistency reliability among people with HIV/AIDS in Vietnam [15] (Cronbach’s alpha = 0.61–0.82 across domains) and people with hypertension in Vietnam (Cronbach’s alpha = 0.65–0.88 across domains) [16].

### Data analysis

IBM SPSS v23 was used for all steps of analysis unless otherwise stated. Little’s test of the expectation-maximization (EM) estimated statistics was used to determine the randomness of missing values (MCAR). A variable with more than 40% of data missing was excluded from further analyses. Complete case analysis (listwise deletion method) was applied.

For objective 1, the COSMIN checklist was used to assess properties of the WHOQOL-BREF_VN_ [17]. For internal consistency, Cronbach’s alpha was calculated for total items and each dimension. For structural validity, CFA was conducted using LISREL 8.8 (maximum likelihood estimation (MLE)) to investigate the loadings of the six domains onto the QOL variable. For construct validity, we conducted known-groups comparison, a univariate ANOVA to evaluate the discriminant function between high (ECOG 1) and low (ECOG 2, 3, 4, or 5) functions.

For objective 2, descriptive statistics were performed, and the percentages of subcategories in overall QOL and general health variables were reported using the midpoint value separating poor and good QOL.

For objective 3, a multinomial logistic regression was performed to identify predictors of HR-QOL score. Step 1 was bivariate analysis of independent variables and dependent variable (HR-QOL) with the Pearson correlation and *p* value reported. Independent variables associated with the QOL in bivariate analysis at *p* ≤ 0.2 in step 1 were retained. Step 2 entailed a correlation matrix to identify pairs of independent variables whose correlation was ≥ 0.8, with subsequent exclusion of the variable with the larger amount of missing data. Step 3 was multinomial logistic regression with backward entry, excluding independent variables *p* > 0.05, rerunning the multinomial logistic regression analysis for the best fit model. Likelihood ratio tests (chi-square, df, and *p*) were used to investigate the model fit. A model was considered a good fit to the data if *p* ≤ 0.05. The best fit model was the last model having a good fit or the model in which all the *p* values of independent variables in the model are < 0.05.

The study was approved by the Institutional Review Boards of Partners Healthcare System in Boston, USA, and the Ministry of Health of Vietnam, and all patients gave written informed consent prior to data collection. The study was performed in accordance with the ethical standards as laid down in the 1964 Declaration of Helsinki and its later amendments.

## Results

### Sample characteristics

We recruited 825 participants, of whom 469 (56.8%) were recruited from the cancer center in the north and 356 (43.2%) from the general hospital in the south.

The sample characteristics are presented in Table 1.

**Table 1:** Sample characteristics (*n* = 825)

Variables	Valid (*n*)	Missing data (*n* (%))	Sample characteristics
			Valid number (percentage) (total 100%)
Study site	824	1 (0.1%)	Cancer center 469 (56.8%) General hospital 356 (43.2%)
Gender	822	3 (0.4%)	Male 441 (53.6%) Female 381 (46.4%)
Type of patients	825	0 (0%)	Inpatient 356 (43.2%) Outpatient 501 (60.7%)
Ethnicity	823	2 (0.2%)	Kinh 807 (98.1%) Others 16 (1.9%)
Region	825	0 (0%)	North 464 (56.2%) Center 9 (1.1%) South 352 (42.7%)
Living location	825	0 (0%)	Rural 454 (55%) Urban 371 (45%)
Education	824	1 (0.1%)	Primary school 188 (22.8%) Secondary school 276 (33.5%) High school 238 (28.9%) College/university 103 (12.5%) Postgraduate 1 (0.1%) Illiterate 18 (2.2%)
Having a paid job	825	0 (0%)	Yes 292 (35.4%) No 533 (64.6%)
Average monthly income	797	28 (3.4%)	Less than 70 USD: 362 (45.4%) 70–120 USD: 155 (19.4%) 120–165 USD: 126 (15.8%) More than 165 USD: 154 (19.3%)
Marital status	823	2 (0.2%)	Single 44 (5.3%) Married 670 (81.4%) Living together as married 3 (0.4%) Separated 24 (2.9%) Divorced 82 (10%) Widowed 0 (0%)
Age	823	2 (0.2%)	Range 20 to 95, mean 56.28, SD 12.24.
Household size	819	8 (0.8%)	Range 0 to 12, median 4
Number of dependent children	822	3 (0.3%)	Range 0 to 9, median 1
Stable relationship with partner	813	12 (1.45%)	Yes 691 (85%) No 122 (15%)
ECOG	824	1 (0.1%)	ECOG 1: 138 (16.7%) ECOG 2: 352 (42.7%) ECOG 3: 206 (25%) ECOG 4: 126 (15.3%) ECOG 5: 2 (0.2%)
Place of care	823	2 (0.2%)	Home 136 (16.5%) Inpatient 378 (45.9%) Day care 2 (0.2%) Outpatient 307 (37.3%) Others 0 (0%)
Stage of cancer	729	96 (11.6%)	Stage 3: 572 (78.5%) Stage 4: 157 (21.5%)
Getting radiotherapy	806	19 (2.3%)	Yes 245 (30.4%) No 561 (69.6%)
Getting chemotherapy	812	13 (1.6%)	Yes 569 (70.1%) No 243 (29.9%)
Years from diagnosis to interview	736	89 (10.78%)	Range 0 to 24, median 1

### Data completeness

There were 1051 (2.7%) missing values. Two variables including primary cancer (missing 417, 50.5%) and metastases (missing 339, 41.1%) were excluded from analysis. Little’s missing completely at random (MCAR) test of expectation-maximization (EM) estimated statistics indicated that missing data were completely at random (chi-square 14.270, df 14, *p* 0.430).

### Objective 1: Evaluate psychometric properties of the WHOQOL-BREF_VN_

For internal consistency, Cronbach’s alpha for total items of the WHOQOL-BREF_VN_ was 0.904. Cronbach’s alphas for each domain were 0.85 (physical), 0.734 (psychological), 0.599 (social relationship), and 0.763 (environmental).

**Fig. 1:**
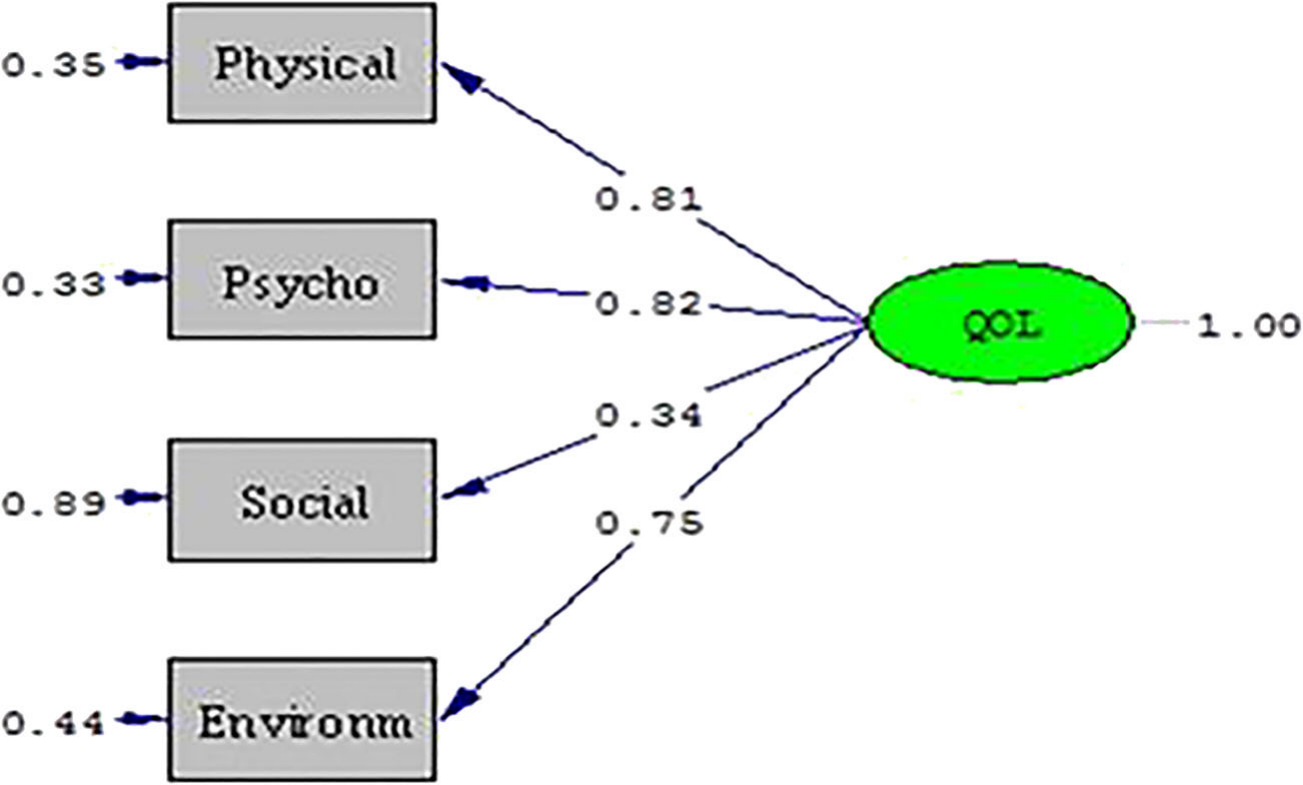
Factor loadings of four domains onto overall QOL item of the WHOQOL-BREF_VN_

With respect to CFA, three (physical, psychological, and environmental) out of four domains are strongly loaded onto the overall QOL item. The loadings were 0.81, 0.82, 0.75, and 0.34 for physical, psychological, environmental, and social relationship domains, respectively. The standardized solution of factor loadings is shown in Fig. 1.

With respect to discriminant validity, all domains significantly discriminated between well and ill patients according to ECOG functional status (*p* < 0.05) (Table 2).

**Table 2:** Discriminant validity of domains of the WHOQOL-BREF_VN_ between well and ill groups according to ECOG physical function

Tests of equality of group means					
	Wilks’ lambda	*F*	df1	df2	Sig.
Physical	.791	215.011	1	815	< 0.01
Psychological	.871	120.951	1	815	< 0.01
Social relationship	.994	5.212	1	815	.023
Environment	.923	68.461	1	815	< 0.01

Wilks’ lambda 0.784, chi-square 197.546, df 4, and *p* < 0.01 demonstrate a statistically significant difference in score prediction for the well and ill groups. The four domains correctly predicted values regarding wellness and illness for 76.3% cases, with accurate predictions being made for 84.7% of well and 74.6% for ill patients.

### Objective 2: Measure of HR-QOL and general health

Regarding the four domains of the WHOQOL-BREF_VN_, median scores of the physical, psychological, social relationships, and environment were 12, 12.67, 13.07, and 12.73, respectively (Table 3). Assuming 12 as the midpoint between bad and good conditions, the participants had neither good nor bad physical, psychological, or environmental conditions and slightly better social relationships.

**Table 3:** Descriptive statistics of the four domains of WHOQOL-BREF_VN_

	Physical	Psychological	Social relationship	Environment
Valid (missing)	822 (3)	820 (5)	824 (1)	821 (4)
Possible range	4–20	4–20	4–20	4–20
Median	12	12.67	13.07	12.73
Mode	12	13	12	15
Standard deviation	2.606	2.199	1.953	2.015
Minimum	4	4	7	6
Maximum	20	20	20	20
Percentiles				
25	9.71	11.33	12	11.5
50	12	12.67	13.33	13
75	13.71	14	14.67	14.5

Concerning HR-QOL, 502 (60.8%) participants perceived neither good nor bad QOL. “Very bad” and “bad” QOL were reported by *n* = 13 (1.6%) and *n* = 175 (21.2%), respectively, while *n* = 127 (15.4%) had “good” and *n* = 8 (1%) had “very good” QOL. Mean and median of HR-QOL were 11.72 and 12, respectively (possible range 4 to 20). Regarding general health, nearly two-fifths (*n* = 324, 39.3%) of the subjects had neutral health status. “Very bad” and “bad” general health were reported by *n* = 44 (5.3%) and *n* = 386 (46.8%), respectively. “Good” and “very good” general health were reported by *n* = 68 (8.2%) and *n* = 3 (0.4%), respectively. The mean and median scores for general health were 10.06 and 8.

### Objective 3: Identify predictors of HR-QOL values

Step 1 (bivariate analyses) identified 9/20 sample characteristics that yielded *p* values > 0.2 (Table 4).

**Table 4:** Bivariate analyses between variables with HR-QOL item (*n* = 825)

Variables	Valid (*n*)	Missing data (*n* (%))	Correlation between variables with HR-QOL item	
			Pearson correlation	*p* (2-tailed)
Study site	824	1 (0.1%)	0.115	0.001
Gender	822	3 (0.4%)	0.002	0.966
Type of patient	825	0 (0%)	0.154	< 0.01
Ethnicity	823	2 (0.2%)	0.041	0.246
Region	825	0 (0%)	0.111	0.001
Living location	825	0 (0%)	0.179	< 0.01
Education	824	1 (0.1%)	0.071	0.041
Having a paid job	825	0 (0%)	− 0.05	0.148
Average monthly income	797	28 (3.4%)	0.162	< 0.01
Marital status	823	2 (0.2%)	0.014	0.686
Age	823	2 (0.2%)	0.104	0.003
Household size	819	8 (0.8%)	0.012	0.733
Number of dependent children	822	3 (0.3%)	−0.104	0.003
Stable relationship with partner	813	12 (1.45%)	0.019	0.587
ECOG	824	1 (0.1%)	− 0.191	< 0.01
Place of care	823	2 (0.2%)	− 0.034	0.324
Stage of cancer	729	96 (11.6%)	− 0.038	0.306
Getting radiotherapy	806	19 (2.3%)	0.014	0.691
Getting chemotherapy	812	13 (1.6%)	0.057	0.103
Years from diagnosis to interview	736	89 (10.78%)	0.01	0.796

The eleven variables were then retained in the correlation matrix which showed that the Pearson correlation between study site and living region was 0.989 (*p* < 0.01, *N* = 824), so the study site was excluded from further analyses. The multinomial logistic regression generated three models (Table 5), of which model 3 was the best fit.

**Table 5:** Likelihood ratio test of the multinomial logistic regression and the percentage of participants for which the models correctly predicted HR-QOL values

Model	Variables	Valid (excluded cases)	Likelihood ratio tests			Percent correct of prediction
			Chi-square	Df	*P*	
Model 1	10 independent variables (type of patient, age, region, living location, education, paid job, monthly income, dependent children, ECOG, chemotherapy)	779 (46)	74.493	76	0.53	61.5
Model 2	6 independent variables (dependent children, type of patient, region, education, paid job, ECOG)	820 (5)	66.958	56	0.15	60.7
Model 3	Dependent children, type of patient, ECOG	821 (4)	35.494	24	0.061	62.2

The best fit model correctly predicted HR-QOL values in 62.2% of participants. The multinomial logistic regression (likelihood ratio test: chi-square 35.494, df 24, *p* 0.061, correct prediction 62.2%) and Pearson correlations revealed that worse HR-QOL was associated with being an inpatient (*p* = 0.015), higher ECOG score (*p* < 0.01), and having dependent children (*p* < 0.01).

## Discussion

This is the first study to measure HR-QOL and its predictors among advanced cancer patients in Vietnam and also the first to evaluate the psychometric properties of the WHOQOL-BREF_VN_ instrument in this population.

We found that the WHOQOL-BREF_VN_ has excellent internal consistency reliability, with Cronbach’s alpha of all items of 0.904 and for the physical (0.85), psychological (0.73), and environmental (0.763) subscales. It had fair internal consistency reliability in the social relationship subscale (0.599).

In terms of CFA, the WHOQOL-BREF_VN_ had excellent structural validity with three out of four domains (physical 0.81, psychological 0.82, and environmental 0.75) strongly loaded onto the HR-QOL item. The social relationship domain played little role in the loading (0.34). The findings are similar to those of the WHOQOL-BREF study, in which the physical domain was the greatest and social relationship the least contributor to the loadings onto the overall QOL item. However, all four domains contributed significantly to explaining observed variance in HR-QOL in prior WHOQOL-BREF studies [18], while social relationships seemed to contribute only slightly in our study. Vietnam has a family-centered cultural tradition. Several generations often live together in the same household, and family members tend to have a strong sense of filial responsibility to care for their parents and relatives [19]. In our study, 81.4% of the patients were married, and the median of the household size was 4 (range 0–12). We suspect that Vietnamese cancer patients have good family support both when they are relatively well and especially when they are sick and thus do not report a change in this domain.

The WHOQOL-BREF_VN_ successfully discriminated between groups with better and poorer functional status (Wilks’ lambda = 0.784, chi-square = 197.546, df = 4, *p* < 0.01), and all the four domains significantly discriminated between these groups (*p* < 0.05) (Table 5).

HR-QOL in our sample (pro-rated to 50/100) was much lower compared to that in two previous studies: the Vietnamese general population (81.6/100), early-stage HIV-infected people (69.3/100), and AIDS patients (65.2/100) [20] and another study of HIV-positive patients on methadone maintenance therapy in Vietnam (59.7, 70, 70.8, and 68.9/100 at baseline, 3 months, 6 months, and 9 months, respectively) [15].

Multinomial logistic regression and the Pearson correlation (*r*) between patient type (*r* = 0.154, *p* < 0.01), number of dependent children (*r* = − 0.104, *p* = 0.003), and ECOG (*r* = − 0.191, *p* < 0.01) indicated that QOL was better among outpatients than inpatients, among patients with few rather than many dependent children, and among patients with a low ECOG score.

Our study had a number of limitations. While we recruited both inpatients and outpatients from both northern and southern regions, convenience sampling and the unknown response rate may have introduced selection bias. In addition, the student interviewers were instructed not to document the primary cancer type or the presence of metastatic disease unless these were certain, and uncertainty was frequent either because the precise diagnosis was not clear or because the interviewer could not determine the diagnosis with certainty. Thus, we were not able to determine the association of cancer type of metastatic disease with the outcomes. However, the results from the MCAR analysis suggest that the missing data did not affect our findings [21]. We also note that this cross-sectional study did not assess test-retest reliability or responsiveness. Also, while the WHOQOL-BREF enables comparison with other clinical populations, face and content validities are not established for advanced disease.

This is the first study to measure HR-QOL of Vietnamese advanced cancer patients. We have demonstrated excellent internal consistency reliability and sound construct and discriminant validity of the WHOQOL-BREF_VN_ in this population.

The majority of participants (77.2%) had acceptable HR-QOL, but a notable minority (22.8%) perceived bad or very bad HR-QOL. All advanced cancer patients should have access to palliative care assessment and interventions. Qualitative studies are needed to articulate the palliative care needs of patients with poor HR-QOL.

Lastly, multinomial logistic regressions revealed that poor ECOG, being an inpatient, and having dependent children were predictors of poor HR-QOL values. Therefore, more attention should be paid to patients having poor ECOG performance status, and social-psychological support should be accessible for inpatients or patients with dependent children.

As cancer-associated suffering and mortality increase in low- and middle-income countries, patient-reported quality of life should be prioritized as an outcome measure for health systems to ensure relief from unnecessary suffering. The WHO has resolved that palliative care is an ethical responsibility of health systems and that universal access to it is necessary to achieve universal health coverage [22]. Reliable measurement of patient-reported quality of life is necessary to assure that palliative care is effective.

## Data Availability

Not applicable.

## Availability of data and material

Data are available from the authors upon reasonable request.

## Standards of reporting

Strobe statement completed.
